# Community Health Seeking Behavior for Suspected Human and Animal Rabies Cases, Gomma District, Southwest Ethiopia

**DOI:** 10.1371/journal.pone.0149363

**Published:** 2016-03-09

**Authors:** Tsegaye Tewelde G/hiwot, Abiot Girma Sime, Benti Deresa, Wubit Tafese, Kifle Weldemichael Hajito, Desta Hiko Gemeda

**Affiliations:** 1 Department of Epidemiology and Biostatistics, College of Public Health and Medical Science, Jimma University, Jimma, Ethiopia; 2 Department of Veterinary Medicine, College of Veterinary Medicine and Agriculture, Jimma University, Jimma, Ethiopia; Shanxi University, CHINA

## Abstract

**Background:**

Timely presentation to appropriate health service provider of sick animals/humans from zoonotic diseases like rabies is important for early case/outbreak detection and management. However, data on community’s health seeking practice for rabies in Ethiopia is limited. Therefore the objective of this study was to determine community’s health seeking behavior on rabies, Southwest Ethiopia.

**Methods:**

A cross-sectional survey was conducted from January 16-February 14, 2015 to collect data from 808 respondents where the respondents were selected using multistage sampling technique. Data were collected using interviewer administered structured questionnaire by trained epidemiology graduate level students. Data were entered to Epidata version 3.1 and analyzed using SPSS version 20 for windows.

**Result:**

Eight hundred three (99.4%) respondents participated in the study. Out of 28 respondents who reported their family members’ exposure to rabies, 8 of them replied that the exposed family members sought treatment from traditional healers. More than nine in ten respondents perceived that humans and domestic animals with rabies exposure should seek help of which 85% of them suggested modern health care facilities as the preferred management option for the sick humans and domestic animals. However, among those who reported sick domestic animals, near to 72% of them had either slaughtered for human consumption, sold immediately, visited traditional healer, given home care or did nothing for the sick domestic animals.

**Conclusion:**

Majority of the respondents had favorable perception of seeking treatment from modern health care facilities for rabies. However, significant number of them had managed inappropriately for the sick domestic animals from rabies. Hence, raising awareness of the community about management of sick domestic animals from rabies and the need for reporting to both human and animal health service providers is needed.

## Introduction

Rabies is an acute viral disease of the central nervous system which affects all warm blooded animals including human beings [1, 2]. Globally, around 3.9 billion people are at risk of contracting rabies and greater than 150 countries are affected [1]. Added to the public health risk, rabies has significant economic impact, the estimated annual cost of rabies is US$ 6 billion with almost US$ 2 billion (~40%) due to lost productivity after premature deaths and a further US$ 1.6 billion spent directly on post-exposure prophylaxis [3].

Rabies is one of the most important public health concerns in Ethiopia in general and in Jimma in particular. Some studies show on average three victims visit Jimma town anti-rabies health centre per day for post-exposure prophylaxis excluding victims going to traditional healers. Thirteen deaths due to rabies were reported from Jimma health center between mid October 2012 and mid January 2013 [4]. In December 2013, a massive outbreak of rabies has occurred and claimed the life of ten individuals in Shabe Sombo district of Jimma zone and the numbers of cases are increasing due to large number of stray dog population in the zone [5].

Integrated reporting of zoonotic diseases is the backbone of surveillance system for early detection and management of outbreaks in both human and animal population [6]. For early detection and management of rabies in humans and animals, integrated human and animal disease surveillance is a crucial one health core approach advocated for developing counties; but this is often not well recognized in these countries. The shortfall is exacerbated by poor infrastructure, limited budget for public health and veterinary sectors, inter-sectoral reluctance to share surveillance data, lack of understanding among national and sub-national decision-makers and stakeholders about the importance of integrated zoonotic disease surveillance. These factors have often led to greater vulnerability to zoonotic diseases emergence and spread as these countries are home to most important zoonotic diseases like rabies [2].

Similar to other developing countries, one sector approach and weak pre-existing animal and human health surveillance system is widely practiced in Ethiopia in general and in Jimma zone in particular. This approach is characterized by delayed outbreak detection and management of rabies in both humans and animal population in southwest Ethiopia. Such case detection is often after massive loss of human and animal lives and probably after occurrence of several outbreaks detected/ undetected in both human and animal populations. Apart from delayed outbreak detection and management, the pre-existing weak surveillance system in the country can jeopardize rabies control and eradication efforts of the sub-Saharan African countries [7].

It is widely recognized that zoonotic diseases control and elimination requires an integrated approach by animal and human health related services [8, 9]. As control efforts progress towards elimination of zoonotic diseases, integrated surveillance is even more critical in order to detect early new cases of those diseases in both human and animal population [9]. Surveillance system using One Health approach is the most effective way of protecting human and animal populations from rabies [10–12]. The feasible and cost-effective surveillance system using One Health approach for major human and animal health problems in developing countries has been strongly advocated in recent years [13]. Ultimately, such approach needs close cooperation of public health and veterinary service providers for effective surveillance of zoonotic diseases [14]. For the better achievement of integrated human and animal surveillance system for rabies, timely presentation to appropriate health service provider of ill animal/human from zoonotic diseases like rabies is important for early case/outbreak detection and management. Because only human and animal health sector integration of surveillance system for zoonotic diseases may not lead to the desired goal unless this integration is known to and practiced appropriately by the community. However, data on community’s health seeking practice for rabies in Ethiopia is limited. Therefore the objective of this study was to determine community’s actual and perceived health seeking behavior on rabies.

## Method and Participants

### Study setting

The research was carried out in Gomma District of Jimma Zone of Oromia Region, Southwest Ethiopia. Agaro is a capital town of Gomma District located at altitude ranging from 1,380 to 1,680 meters above sea level. However, some points along the southern and western boundaries have altitudes ranging from 2,229 to 2,870 meters. The projected total population of the district in 2014 from the 2007 national census is 246, 381 residing in 51,652 households. Among which 3, 094 (5.99%) are urban households and 48,558(94.01%) are rural households [15] residing in 36 rural and 3 urban kebeles (Kebele is the smallest administrative unit in Ethiopia) [16]. The study was conducted from January 16-February 14, 2015.

### Study design

A cross-sectional study design was employed.

### Study Population

The study populations were heads of the households who resided in the district for at least 6 months and lives in residential houses, however, mentally incapable household heads and refusals were excluded from the study.

### Sampling procedure

The sample size was determined using single population proportion formula by considering the following parameters: a 50% proportion (p) of households sought modern health facility for suspected human and animal rabies, 5% level of significance (α), and 5% margin of error (d). Considering design effect (DEff = 2) and 5% non-response rate, the final sample size was 808 households. Multi-stage cluster sampling stratified in to urban and rural Kebeles was used to select primary (Kebele) and secondary (households) sampling units. Thirty percent of urban (n = 1) and rural (n = 11) kebeles were selected randomly. Using stratified proportionate to size allocation, forty eight and seven hundred sixty households from the selected urban and rural kebeles were selected respectively. Sampling frame was prepared for the urban and rural households using house number and then simple random sampling technique was employed to select household for the interview. Head of the selected household was interviewed on perceived and experienced human and animal rabies health seeking behavior.

### Measurement

A pretested structured questionnaire was used to collect data on general, actual and perceived health seeking behavior of the households. Actual health seeking behavior of the households for suspected rabies in humans and animals was assessed by asking where the household sought treatment/care for the victim family members, domestic animals (cat, dog, cattle, sheep, goat, camel, chicken, horse, mule and donkey) from rabies. Perceived health seeking behavior of households, with and without domestic animals, for suspected rabies in humans and animals was also assessed by asking what should be done if family member and/or domestic animal were bitten by suspected rabid animal. Data were collected by four trained graduate program students in Veterinary and Public Health Epidemiology from Jimma University. Data collection process was closely supervised by two Veterinary and Public Health Epidemiologists.

### Data analysis

Data were checked and edited for completeness then entered into Epidata version 3.1 and finally analyzed using SPSS version 20.0. Descriptive analysis like frequency and percentage was run to check outliers, missing data and inconsistencies. Respondents’ socio-demographic characteristics, their actual and perceived health seeking practices were analyzed using relative frequency statistics and absolute number. Actual and perceived Health seeking practices were categorized as: visiting modern health facility, visiting traditional healers, doing nothing, and managing at home (slaughtering for consumption, selling, and nursing at home). Summary statistics (percentage) was computed for each of the aforementioned categories. Modern health facilities-includes all animal and human health institutions (hospitals, health centers, clinics, health posts and veterinary clinics). Suspected human/animal rabies cases were assessed by yes/no oral response of respondents.

### Human and Animal Ethical Considerations

Ethical clearance was obtained from both Health Sciences College and School of Veterinary Medicine Institutional review Board of Jimma University. For the details please see S1 and S2 Text. Consent form was developed by the research team and approved by the Institutional review Board of Jimma University. Permission letter was sought from both human and animal Gomma district health offices to conduct the research. Before data collection, the objective of the study was explained to the participants and data collection was commenced only after obtaining written consent. Similarly the information was handled confidentially and it was used only for research purpose.

## Results

### Socio-demographic and economic characteristics

Eight hundred three (99.4%) respondents with mean age of 40.1±10.5 years participated in the study. The majority of them were: male (63.8%), rural residents (94%), farmer in occupation (49.3%), literate (70%), Islam in religion (72.2%) and had children (85.4%) Table 1.

**Table 1:** Socio-demographic and economic characteristics of respondents in Gomma district, Southwest Ethiopia, January 16-Februay 14, 2015 (N = 803).

Variable	Categories	Frequency	Percentage
**Age in years**	18–27	91	11.3
	28–37	239	29.8
	38–47	312	38.9
	48–57	112	13.9
	58–86	49	6.1
**Residence**	Rural	755	94.0
	Urban	48	6.0
**Educational status**	Illiterate	241	30
	Primary education	376	46.8
	Secondary education	106	13.2
	Above secondary	80	10
**Occupational status**	Farmer	396	49.3
	Businessmen/women	143	17.8
	Housewife	125	15.6
	Employed	61	7.6
	Daily laborer	38	4.7
	Other	40	5
**Religion**	Islam	580	72.2
	Orthodox	173	21.5
	Protestant	47	5.9
	Other	3	0.4
**Ethnicity**	Oromo	523	65.1
	Guraghe	93	11.6
	Amhara	74	9.2
	Others	113	14.1
**Marital status**	Married	680	84.7
	Single	87	10.8
	Widowed	33	4.1
	Separated	3	0.4
**Had child**	Yes	686	85.4
	No	117	14.6
**Personal income**	Yes	549	68.4
	No	254	31.6
**Household monthly income in Birr**	1^st^ quartile (8–350)	201	25.0
	2^nd^ quartile (351–720)	201	25.0
	3^rd^ quartile (721–1200)	210	26.2
	4^th^ quartile (>1200)	191	23.8

### General health seeking behavior for sick family members and domestic animals

Four hundred ninety one (61.1%) respondents reported that there was a sick family member in the past 12 months prior to data collection period of which 46% of them stated malaria as a perceived cause of sickness. More than three-fourth (77%) of sick members sought treatment from health center, however,16.2% sought treatment from traditional healer, religious institution, homecare and didn’t sought any care Table 2.

**Table 2:** General health seeking behavior of households for sick family members and domestic animals in Gomma district, Southwest Ethiopia, January 16-Februay 14, 2015.

		Yes	No
Variables	Categories	Frequency (%)	Frequency (%)
**Sick family member in the past 12 months (N = 803)**		491(61.1)	312(38.9)
**Perceived cause of sickness (N = 491)**	Malaria	226(46)	265(54.0)
	Diarrheal diseases	139(28.3)	352(71.7)
	Respiratory problem	84(17.1)	407 (82.9)
	Skin problem	42(8.6)	449(91.4)
	Tuberculosis	37(7.5)	454(92.5)
	Do not know	102(20.8)	389(79.2)
**Sought treatment from (N = 491)**	Hospital	79(16.1)	412(83.9)
	Health center	378(77.0)	113(23)
	Health post	3(0.6)	488(99.4)
	Private clinic	60(12.2)	431(87.8)
	Traditional healer	24(4.9)	467(95.1)
	Religious institution	14(2.9)	477(97.1)
	Home care treatment	7(1.4)	484(98.6)
	Nothing done	17(3.5)	474(96.5)
**Had any domestic animal within 12 months (N = 803)**		604(75.2)	199(24.8)
**Domestic animal sick within 12 months (N = 604)**		283(46.9)	321(53.1)
**Perceived cause of disease (N = 283)**	Brucellosis	27(9.5)	256(90.5)
	Anthrax	24(8.5)	259(91.5)
	Rabies	11(3.9)	272(96.1)
	Have no idea	229(81)	54(19)
**Sought treatment from (N = 283)**	Veterinary clinic	191(67.5)	92(32.5)
	Traditional healer	6(2.1)	277(97.9)
	Home care treatment	7(2.5)	276(97.5)
	Slaughtered for human consumption	15(5.3)	268(94.7)
	Immediately sold	9(3.2)	274(96.8)
	Immediately killed	2(0.7)	281(99.3)
	Nothing done	34(12)	249(88)
	Have no idea	20(7.1)	263(92.9)

Three-fourth of the respondents reported that their family had domestic animal within the past 12 months prior to data collection period of which 46.9% of them reported that they had sick domestic animal. With regard to the perceived cause of sick domestic animals, 81% have no idea, 8.5% mentioned anthrax and 3.9% rabies as the perceived causes of the diseases. One hundred ninety one (67.5%) of the families who had sick domestic animal reported that their family had visited Veterinary clinic to seek treatment. Close to 30% of the respondents, however, either slaughtered for human consumption, immediately sold it, did nothing or had no idea about what to do for the sick domestic animal Table 2.

### Actual health seeking behavior for rabies in human and domestic animals

More than nine in ten of the respondents heard about rabies. Twenty eight respondents reported their family members were exposed to rabid dog in the past 12 months and eight of them received treatment from traditional healers Table 3.

**Table 3:** Respondents’ actual health seeking behavior for rabies in human and domestic animals, Gomma district, Southwest Ethiopia, January 16-Februay 14, 2015.

		Yes	No
Variables	Categories	Frequency (%)	Frequency (%)
**Heard about rabies (N = 803)**		775(96.5)	28(3.5)
**Family members exposed/bitten by rabid dog in the past 12 months (N = 775)**		28 (3.6)	747(96.4)
**Sought treatment from (N = 28)**	Hospital	24(85.7)	4(14.3)
	Traditional healer	8(28.6)	20(71.4)
	Health center	7(25.0)	21(75.0)
	Health post	1(3.6)	27(96.4)
**Any domestic animal victim of rabies within 12 months(N = 604)**		11(1.8)	593(98.2)
**Sought treatment from (N = 11)**	Veterinary clinic	3(27.3)	8(72.7)
	Slaughtered for human consumption	2(18.2)	9(81.8)
	Traditional healer	1(9.1)	10(90.9)
	Home care treatment	1(9.1)	10(90.9)
	Nothing done	2(18.2)	9(81.8)
	Have no idea	2(18.2)	9(81.8)

Eleven respondents reported their domestic animals were sick from rabies within the past 12 months prior to data collection period. But only 27% of respondents received treatment from veterinary clinic for their domestic animals sick from the disease while the remaining respondents, however, either slaughtered for human consumption, immediately sold, did nothing, went to traditional healer, gave home care, had no idea about what was done for the sick domestic animals Table 3.

### Perceived health seeking behavior for rabies in human and domestic animals

More than 98%of the respondents perceived that their family members should seek help when bitten by suspected rabid animal among which85.3% of family members perceived that modern health facilities were the best places for the treatment of rabies Table 4.

**Table 4:** Respondents’ perceived health seeking behavior for rabies in human and domestic animals, Gomma district, Southwest Ethiopia, January 16-Februay 14, 2015.

		Yes	No
Variables	Categories	Frequency (%)	Frequency (%)
What should be done if family member bitten by suspected rabid animal	Seek help	795(99)	8(1)
What do you think is the management option for	Visiting modern health facilities	678(85.3)	117(14.7)
rabies (N = 795)	Visiting Traditional healers	201(25.3)	594(74.7)
	Visiting Religious institutions	10(1.3)	785(98.7)
	Have no idea	1(0.1)	794(99.9)
What should be done if domestic animal is bitten by suspected rabid animal(N = 803)	Seek help	791(98.5)	12(1.5)
What do you think is the management option for	Visiting modern health facilities	706(89.3)	85(10.7)
rabies (N = 791)	Visiting traditional healers	108(13.7)	683(86.3)
	Slaughtering for human consumption	53(6.7)	738(93.3)
	Immediately considering for selling	17(2.1)	774(97.9)
	Visiting religious institutions	10(1.3)	785(98.7)
	Immediately killing	5(0.6)	786(99.4)
	Home care treatment	2(0.3)	789(99.7)
	Have no idea	27(3.4)	764(96.6)
**Do you think that domestic animal died of rabies should be reported (N = 803)**		482(60)	321(40)
**To whom it should be reported (N = 482)**	Animal health care provider	289(60)	193(40)
	Human health care provider	50(10.4)	432(89.6)
	One to five (1:5) development army	107(22.2)	375(77.8)
	Kebele administrator	103(21.4)	379(78.6)

Near to ninety nine percent of respondents perceived that domestic animals sick from rabies should get help of whom more than 89% perceived that modern health facilities are the best places for treatment. However, 28% of the respondents perceived that either slaughtering for human consumption, immediately selling, visiting traditional healer or giving home care are the best management options for the sick domestic animals from rabies. Two out of five respondents perceived that domestic animals died of rabies should not be reported. Among those respondents who perceived that domestic animals died of rabies should be reported, 60% of them preferred animal health care providers for reporting of animals died of rabies Table 4.

## Discussion

Forty seven and sixty one percent of the respondents reported that their family members and their domestic animals were sick in the past 12 months respectively for which more than two-third of them received treatment from modern health care facilities for the sick family members and domestic animals respectively. Twenty eight (3.6%) of the respondents reported that their family members were sick from rabies in the past 12 months while eleven (1.8%) reported that their domestic animals were sick from the disease. However, eight of rabies exposed humans received treatment from traditional healers. More than nine in ten of the respondents perceived that both human and domestic animals that had exposure to suspected rabies cases should seek help. Even though more than 85% of the respondents suggested visiting modern health care facility as the preferred management option for the sick humans and domestic animals from rabies, seven in ten of the respondents who had sick domestic animals had either slaughtered for human consumption, sold immediately, visited traditional healer, gave home care or did nothing for the sick domestic animals from rabies. Sixty percent of the respondents believed that animals died of rabies needs to be reported to animal health care providers.

Twenty eight (3.5%) respondents reported that their family members were bitten by suspected rabid dogs in the past 12 months; which is lower than the finding in Tanzania (8%) [17], and in rural Bangladesh (7.3%) [18]. The difference could be explained by the fact that the current study collected data on 12 months experience of bite from suspected rabid dog while the other studies collected data on a lifetime experience of suspected rabid dog bite. On the other hand, eleven (1.8%) respondents reported that their domestic animals were bitten by suspected rabid animal in the past 12 months. This implies that from every 1, 000 human and animal population, there would be 35 and 18 rabid human and domestic animal cases in 12 months; given poor actual health seeking behavior in this population could mean massive outbreaks and fatalities from the disease.

It was also identified that eight (28.6%) of rabies exposed humans received treatment from traditional healers in the past 12 months. This is much better than the finding (90%) reported from rural Bangladesh [18]. The difference may be due to the fact that the study in Bangladesh was carried out 9 years back as compared to the current one. This implies that there is still societal reliance on traditional healers for rabies treatment in Ethiopia which could lead to under reporting of the magnitude of the diseases and hence sub-optimal planning for human rabies management, delayed case detection, preventable outbreaks and death from rabies. Similarly eight (72.7%) of the respondents who reported that their domestic animals were exposed to rabies in the past 12 months had either visited traditional healers, slaughtered the animal for human consumption, given home care or done nothing for the exposed domestic animals. This would promote circulation of rabies disease to other domestic animals, humans in close contact and wild animals. This could easily contribute for the occurrence of rabies outbreak and seriously reduce the flow of tourists to the affected area.

The majority (85%) of the respondents perceived that both human and domestic animals that had exposure to rabies should seek help; this is consistent with the finding from Tanzania (90%) [17] and Sri Lanka (96%) [19]. Similarities of the findings could be as a result of comparable socio-demographic characteristics of respondents and rabies epidemiology in the studied areas of these countries. This implies that such favorable treatment seeking perception of community can play crucial role for the prevention and control efforts of rabies in the district.

Moreover, 60% of the respondents believed that animals died of rabies needs to be reported to animal health care providers. This will facilitate for early rabies case/outbreak detection by health care practitioners, prevention and control implementation efforts and policies. There could be over or under estimation of rabies cases that arises from oral reports of respondents and hence interpretation of the findings should be made with this limitation.

## Conclusion

More than two-third of the respondents in general had received treatment from modern health care facilities for the sick family members and domestic animals for various ailments in the past 12 months. Close to 30% of rabies exposed humans received treatment from traditional healers. Even though more than 85% of the respondents suggested visiting modern health care facility as the preferred management option for the sick humans and domestic animals from rabies, near to seven in ten of the respondents who had sick domestic animals had either slaughtered for human consumption, sold immediately, visited traditional healer, gave home care or did nothing for the sick domestic animals from rabies. Sixty percent of the respondents believed that animals died of rabies needs to be reported to animal health care providers. Hence, raising awareness of the community about management of sick domestic animals and humans from rabies and the need for reporting to both human and animal health service providers is needed.

## Supporting Information

S1 Text Ethical clearance involving human study subjects obtained from Health Sciences College IRB of Jimma University. (PDF)

S2 Text Ethical clearance involving animal study subjects obtained from School of Veterinary Medicine IRB of Jimma University. (PDF)

S3 Text Funding statement on community health seeking behavior for suspected rabies cases. (DOCX)

S4 Text Related manuscript on community health seeking behavior for suspected anthrax cases. (DOCX)
