# Supplementary material for: Community Health Seeking Behavior for Suspected Human and Animal Rabies Cases, Gomma District, Southwest Ethiopia
Source: PLoS One. 2016 Mar 9;11(3):e0149363. doi: 10.1371/journal.pone.0149363 (PMC4784896; doi:10.1371/journal.pone.0149363)
Supplement: S1 Text — (PDF) [file pone.0149363.s001.pdf]

JIMMA UNIVERSITY
ጅማ ዩኒቨርሲቲ

ቁጥር
Ref.No RPGC/522/2014
ቀን
Date 30/10/2014

To: - Mr. Abiyot Girma
- Mr. Tsegaye Tewolde
- Dr. Benti Deresa
- Dr. Wubit Tafese
- Mr. Desta Hiko

College of Public Health and Medical Science
Jimma University

Subject: *Outcome of Ethical Review of Your Research Plan*

This is to acknowledge an amended ***" Integrated rabies and anthras surveillance system using one health approach: Knowledge and practice gap in Gomma district of Jimma Zone, southwest Ethiopia."*** has been reviewed by the ethical clearance board of Jimma University, according to the standardized principle and a procedure which is designed in line with the national and WHO guidelines.

As previously approved, currently with pleasure we inform you that the project is ethically approved and thus you are requested to implement the research plan as per the approved protocol.

Sincerely,

Dr. Beyene Wondafrash (Ass Prof.)
Health Research & Post Graduate Coordinator

CC:
- Ethical Review Board
Jimma University

Tel. +251-47 11 114 57 PBX:+251471111458-60 Fax: +2514711111450 +251471112040 P.O.Box. 378 JIMMA,ETHIOPIA E-mail:ero@ju.edu.et website:http://www.ju.edu.et
