# Supplementary material for: Community Health Seeking Behavior for Suspected Human and Animal Rabies Cases, Gomma District, Southwest Ethiopia
Source: PLoS One. 2016 Mar 9;11(3):e0149363. doi: 10.1371/journal.pone.0149363 (PMC4784896; doi:10.1371/journal.pone.0149363)
Supplement: S2 Text — (PDF) [file pone.0149363.s002.pdf]

JIMMA UNIVERSITY
ጅማ ዩኒቨርሲቲ

ቁጥር
Ref.No. Vet.med/19/2014
ቀን
Date 07/11/2014

From: School of Veterinary Medicine
To: Mr. Abiot Girma
Mr.Tsegaye Tewolde
Dr.Benti Deresa
Dr.Wubit Tafese
Mr.Desta Hiko
JUCAVM

Subject: Outcome of Ethical review of your research plan

This is to acknowledge an amended"***Integrated rabies and anthrax surveillance system using one health approach*: *knowledge and practice gap in Gomma district of Jimma Zone, Southwest Ethiopia***" has been reviewed by the ethical clearance board of school of veterinary medicine of Jimma University which is in line with the national and international research ethics guideline that involves human/animal as research subjects.

The Institution Review Board (IRB) of veterinary medicine of Jimma University is glad to inform you that the project is ethically approved and thus you are requested to implement the research plan as per the approved protocol.

Sincerely,

Haileu Degefu (Dr.)
School of Veterinary Medicine Head

CC
Ethical review board
JUCAVM

Contact Us: Tel +251 – 47-111-54-05 Fax +251- 47-111-09-34

Tel. +251-47 11 114 57
PBX: +251471111458-60
Fax: +2514711114 50
+251471112040
P.O.Box. 378
JIMMA, ETHIOPIA
E-mail: ero@ju.edu.et
website: http://www.ju.edu.et
