# Supplementary material for: Community Health Seeking Behavior for Suspected Human and Animal Rabies Cases, Gomma District, Southwest Ethiopia
Source: PLoS One. 2016 Mar 9;11(3):e0149363. doi: 10.1371/journal.pone.0149363 (PMC4784896; doi:10.1371/journal.pone.0149363)
Supplement: S3 Text — (DOCX) [file pone.0149363.s003.docx]

**Funding Statement**

The source of financial fund was from one health central and eastern Africa (OHCEA) with a grant number of 0714/16 / Surveillance System-16 For a research project on “**Integrated rabies and anthrax surveillance system using one health approach: Knowledge and Practice gap in Gomma district of Jimma zone, Southwest Ethiopia”. The website of the OHCEA is ohcea**.org and Professor Kifle weldemichael was contracted by OHCEA as local supervisor of the research team and he was involved during designing, data collection and critically reviewing the manuscript. Hence, we considered him as an author in this manuscript

# 
