# Supplementary material for: Community Health Seeking Behavior for Suspected Human and Animal Rabies Cases, Gomma District, Southwest Ethiopia
Source: PLoS One. 2016 Mar 9;11(3):e0149363. doi: 10.1371/journal.pone.0149363 (PMC4784896; doi:10.1371/journal.pone.0149363)
Supplement: S4 Text — (DOCX) [file pone.0149363.s004.docx]

# Community Health Seeking Behavior for suspected Anthrax cases in humans and animals, Gomma district, Southwest Ethiopia

Abiot Girma^1^, Tsegaye Tewelde^2§^, Desta Hicko^3^, Benti Deresa^5^, Webit Tafese^6^, Kifle Weldemichael^4^

^1-4^Department of Epidemiology and Biostatistics, College of public health and medical science, Jimma University, Jimma, Ethiopia

^5,6^Department of Veterinary medicine, college of veterinary medicine and agriculture, Jimma University, Jimma, Ethiopia

^1§^Corresponding author

Abiot Girma: [abiot.girma@yahoo.com](mailto:abiot.girma@yahoo.com)

Tsegaye Tewelde: [tsegaye.tewelde@yahoo.com](mailto:tsegaye.tewelde@yahoo.com)

Desta Hicko: destahiko@gmail.com

Benti Deresa: bentijc@gmail.com

Wubit Tafese: [wubit.tafese@yahoo.com](mailto:wubit.tafese@yahoo.com)

Kifle Weldemichael: bethy_kifle@yahoo.com

# Abstract

**Background**: timely presentation to appropriate health service provider of sick animals/humans from zoonotic diseases like rabies and anthrax is important for early case/outbreak/ detection and management. However, data on community’s health seeking practice for anthrax in Ethiopia is limited. Therefore the objective of this study was to determine community’s health seeking behavior for suspected human and animal anthrax cases, Gomma district, Southwest Ethiopia

**Methods:** A cross-sectional survey was conducted from January 16-February 14, 2015 to collect data from 808 respondents where the respondents were selected using multistage sampling technique. Data about community health seeking behavior was collected using interviewer administered structured questionnaire by trained veterinary and public health epidemiology graduate level students. Edited data were entered to Epidata version 3.1 and analyzed using SPSS version 20 for windows.

**Result:** Eight hundred three (99.4%) respondents with mean age of 40.1±10.5 years participated in the study. Two respondents reported that their family members were victims of anthrax for which both of them sought treatment from hospital. More than nine in ten of the respondents perceived that humans and domestic animals suspected of anthrax should seek help; 85% of them suggested modern health care facilities as the preferred management option for the sick humans and domestic animals. However, among the respondents who reported sick domestic animals, half of them had either slaughtered for human consumption, sold immediately, visited traditional healer, given home care or did nothing for the sick domestic animals. In addition 60% of the respondents believed that animals died of both rabies and anthrax needs to be reported to animal health care providers only.

**Conclusion:** The majority of the respondents had favorable perception of seeking treatment from modern health care facilities for zoonotic diseases. However, significant number of them had either slaughtered for human consumption, sold immediately, visited traditional healer or given home care for the sick domestic animals. Hence, raising awareness of the community about management of sick domestic animals and the need for reporting to both human and animal health service providers is needed.

**Keywords**: Community, health seeking behavior, anthrax, integrated surveillance, one health approach, Ethiopia
